# Spiritual Healing

**Published:** 1911-09-23

**Authors:** 


### Spiritual Healing.

After a painstaking and conscientious inquiry, the committee appointed two years ago by the British Medical Association to investigate healing by spiritual means has issued its report. It is perhaps not to be expected that those who have exploited the name and teachings of Christ for personal ends will accept the conclusions which the committee has published; but it will be disappointing if those who have honestly believed and practised mental suggestion for the relief of sickness should fail to appreciate the importance of the report and the sincerity of those who have compiled it. Anyone who will take the trouble to read this document will find himself convinced, whether or not he agrees with all its clauses, of the earnestness and thoroughness with which the committee has applied itself to the work of eliciting every fact bearing on the subject of "spiritual healing." In promoting inquiries such as this and in publishing the report the Association is carrying on a work which is to the advantage both of the profession and the public.

The reference of the matter to the committee originated primarily from the somewhat indefinite kind of recognition of clerical participation in therapeutics which was discussed at the Lambeth Conference and elsewhere. While the accredited leaders of the Church were distinctly shy of committing themselves to any attitude beyond one of benevolent neutrality, there were sundry enthusiasts who claimed much more for "spiritual healing" than either the medical profession, the Lambeth Conference, or ordinary common-sense could possibly admit. To these gentlemen the British Medical Association's committee applied for full particulars of cases, with a view to full investigation. Great difficulty was, however, encountered in getting details and following up the cases; in fact, the only cases actually forthcoming were provided by the "Society of Emmanuel." Ten patients were thus personally examined, but opportunity for subsequent examination, though requested, was not permitted. Even details in writing of cases were but scantily to be obtained, and in fact a marked disinclination to help the committee at all seems to have been manifested in some quarters. This shyness may have, no doubt, originated with the patients themselves, not with their "healers"; or may have been due to distrust of the doctors on the committee by the latter. But it is incumbent on those who fancy they have made discoveries which will benefit mankind to court publicity and to prove their case in the most open and above-board manner if they wish to be taken seriously and to be treated as honest men.

The Bishop of Winchester, who was chairman of the committee of the Lambeth Conference which dealt with this question, was invited to state his views, and it is refreshing to note the robust common-sense with which he replies. Substantially his opinions differ little from the conclusions set forth in the committee's report. Thus the first paragraph of the latter sets forth that there is no difference in kind beween "spiritual healing" and other forms of mental, psychic, or faith healing. Dr. Ryle, indeed, goes almost a shade further, for he draws no distinctions between Mahomedans, Christian Scientists, Church of England healers, and members of other religions. The essential factor, proceeds the report, in all forms of psychic healing is mental suggestion; there is abundant evidence of the efficacy of this in the treatment of many diseases, but no evidence has been procured by the committee of any authenticated cure of organic disease. The benefits of such hypnotic suggestion can be obtained from qualified medical practitioners, whose training enables them to distinguish the conditions which are amenable to this kind of therapy from those which should be dealt with by other kinds of medical or surgical treatment. In this excellent sentence the committee expressly declares that suggestion is a part of recognised medical treatment, a fact patent enough to every medical man, but not always comprehended by the laity. Finally, the formal co-operation of the clergy with medical men is deprecated on the grounds that all the benefits which may accrue to the sick from the ministrations of the former may be obtained as things are at present, a conclusion as full of plain sense as all the rest of this interesting and valuable report.

